# Enhancing Jabon (*Anthocephalus cadamba*) Laminated Board Properties with Impregnation of Citric Acid, Boric Acid, and Polystyrene

**DOI:** 10.3390/polym17172367

**Published:** 2025-08-30

**Authors:** Rudi Hartono, Raynata Andini Br Tarigan, Muhammad Navis Rofii, Ihak Sumardi, Aprilia Kartikawati, Jajang Sutiawan, Falah Abu, A. M. Radzi

**Affiliations:** 1 Forest Products Department, Faculty of Forestry, Universitas Sumatera Utara, Medan 20155, Indonesia; raynataandini@gmail.com; 2 Faculty of Forestry, Universitas Gadjah Mada, Yogyakarta 55281, Indonesia; navis_r@ugm.ac.id; 3 School of Life Sciences and Technology, Institut Teknologi Bandung, Bandung 40132, Indonesia; ihak@itb.ac.id; 4 Research Center for Biomass and Bioproducts, National Research and Innovation Agency, Cibinong 16911, Indonesia; apri025@brin.go.id (A.K.); jaja007@brin.go.id (J.S.); 5 Faculty of Applied Sciences, Universiti Teknologi MARA (UiTM), Shah Alam 40450, Selangor, Malaysia; falah@uitm.edu.my; 6 Smart Manufacturing Research Institute (SMRI), Universiti Teknologi MARA (UiTM), Shah Alam 40450, Selangor, Malaysia; 7 Institute of Tropical Forestry and Forest Product (INTROP), Universiti Putra Malaysia (UPM), Serdang 43400, Selangor, Malaysia; mohdradzi@upm.edu.my

**Keywords:** impregnation, modification, boric acid, citric acid, polystyrene, laminated board

## Abstract

A good way to produce large-sized wood products from small-diameter logs is by using laminated boards. The lamina undergoes an impregnation pretreatment to improve its quality before being formed into laminated boards (LBs). This research was performed to analyze the effects of an impregnation treatment on Jabon lamina with citric acid, boric acid, and polystyrene solutions on the physical and mechanical properties of Jabon LB. The Jabon lamina was first pretreated with citric acid, boric acid, and polystyrene by vacuuming for 30 min and pressing for 30 min at a pressure of 6.6 bar. The laminas were glued using isocyanate adhesive with a spreading rate of 280 g/m^2^, consisting of three layers, which were cold pressed for 24 h. LB’s physical and mechanical properties were affected by the nature of the impregnating agent. Impregnating the lamina with citric acid and boric acid increased the density and moisture content of the laminated board, decreasing its mechanical properties. On the contrary, polystyrene-impregnated LB improved. After soaking in hot water, no LB displayed delamination, indicating high bonding performance. The best impregnating agent for lamina pretreatment was polystyrene, followed by boric acid and citric acid. The chemical compound, functional group, and degree of crystallinity of treated Jabon LB all changed due to the impregnation process.

## 1. Introduction

Plantation forest development is expected to increase supply and meet the wood requirements of the wood industry [1]. Plantation forests are generally planted with fast-growing species with short harvesting rotations to ensure a stable supply of raw materials for the wood industry. Among these species, Jabon (*Anthocephalus cadamba*) has attracted attention due to its rapid growth, a simple planting method, and simple processing characteristics [2]. Jabon wood has an average specific gravity of 0.35–0.55 g/cm^3^ and is classified as strength class III [3]. Although Jabon wood is known for having fairly strong tensile, compressive, and flexural strength, it has weaknesses when used as furniture and construction materials. Jabon has several limitations that restrict its application, including low density, high hygroscopicity, poor dimensional stability, and vulnerability to biological degradation. These limitations make it unsuitable for structural applications without modification. As a result, laminated boards were developed to utilize fast-growing wood and produce better wood products [4].

Laminated boards enable versatile manufacturing processes from small material sizes into product shapes and sizes according to furniture demands and preferences [5], combining wood and non-wood materials to create panel products [6]. Furthermore, laminated boards can efficiently utilize raw materials, including waste from the sawmill industry. According to previous research, sawmill waste—such as durian, plajau, and surian bawang wood—can make laminated beams and boards using polyvinyl acetate (PVA) adhesive. The findings demonstrated that the laminated board in layers two and three and the laminated beam in layers three and four have been approved by the Indonesian National Standard 010608-89 [7]. In other words, laminated wood can be produced from low-grade timber, reducing the costs associated with high-quality wood. Fast-growing species have been used as raw materials for laminated boards, including sengon and bayur wood [8], sengon and merbau wood [9], sengon and petung bamboo [10], sengon and coconut wood [11], sengon, Jabon merah, acacia hybrid, and bamboo [12], and sengon and Jabon [13]. Using fast-growing wood has several disadvantages, such as poor dimensional stability and susceptibility to wood-destroying organisms. Therefore, wood lamina needs to be enhanced in quality before being utilized for manufacturing laminated boards.

Various wood modification methods have been developed and widely applied to improve physical and mechanical performance. Well-established approaches include acetylation, which reduces hygroscopicity through the esterification of hydroxyl groups; furfurylation, which enhances dimensional stability and biological resistance by in situ polymerization of furfuryl alcohol; and thermal modification, which improves durability but often at the expense of mechanical strength [14,15]. More recent strategies involve the incorporation of nanocellulose and other nanoscale reinforcements to improve strength and interfacial bonding in wood composites [16,17]. While these treatments are effective, they also present challenges such as cost, process complexity, or potential environmental concerns.

Impregnation treatment has been widely recognized as an effective method for improving the performance of fast-growing and low-density wood species by introducing chemical agents deep into the cell walls and lumina under vacuum and/or pressure. The mechanism depends on the nature of the impregnating agent: citric acid, a polycarboxylic acid, promotes esterification reactions with hydroxyl groups in cellulose and hemicellulose, reducing hygroscopicity and improving dimensional stability while potentially weakening mechanical strength at high concentrations due to hydrolysis [18,19]. Boric acid, a traditional water-soluble preservative, forms stable borate–polyol complexes with cell wall carbohydrates, which enhances fire resistance and biological durability, although it can also reduce strength if overdosed [20,21]. In contrast, polystyrene undergoes in situ polymerization within lumina and cell wall cavities, forming hydrophobic networks that fill voids and reinforce wood microstructure, thereby improving both mechanical properties and dimensional stability [22,23]. These distinct mechanisms illustrate how different chemical pathways—esterification, complexation, and polymer reinforcement—can be strategically applied to upgrade low-grade wood species.

According to previous studies, modification of belangke bamboo lamina by immersion for 6–24 h in citric acid, boric acid, and polystyrene solutions can enhance the physical and mechanical properties of belangke bamboo laminated boards [24]. Meanwhile, the reactivity and effectiveness of these three chemical solutions on wood, especially fast-growing Jabon wood, have never been studied. In addition, there have been limited studies on combining eco-friendly chemicals (citric acid and boric acid) with synthetic polymers (polystyrene) in Jabon laminated boards. The choice of these impregnating agents is justified by their distinct mechanism effects on wood. However, sustainability issues with polystyrene have been acknowledged, and it has been emphasized that this study explores both potential benefits and limitations compared with bio-based alternatives. Furthermore, compared to immersion in a prior study, impregnation treatment produces more uniform and deeper penetration by forcing treatment materials deeply into the wood’s cellular structure using vacuum and/or pressure [25]. Therefore, this study aims to analyze the effects of impregnation treatment on Jabon lamina with citric acid, boric acid, and polystyrene solutions on the mechanical and physical properties of Jabon laminated boards.

## 2. Materials and Methods

### 2.1. Materials

Jabon wood (*Anthocephalus cadamba*) was obtained from Paya Geli, North Sumatra, Indonesia. The boards were prepared with the grain oriented parallel to the longitudinal axis. The average density of the Jabon wood was approximately 0.35–0.4 g/cm^3^, and the initial moisture content was 10%. The samples for laminated board production were cut into lamina with dimensions of 30 cm × 2.5 cm × 0.5 cm. Citric acid, boric acid, and polystyrene were used as impregnating agents for the Jabon laminae, while laminated board fabrication employed an isocyanate-based adhesive. All chemicals were of analytical grade and obtained from PT. Digdaya Indo Karya (Bogor, Indonesia), and the adhesive was supplied by PT. Polychemie Asia Pacific Permai (Jakarta, Indonesia).

### 2.2. Sample Preparation

Jabon wood was converted into lamina with length, width, and thickness dimensions of 30 cm × 2.5 cm × 0.5 cm. The Jabon lamina was dried in an oven at 60 °C for 3 days to ensure uniform moisture content. Then the dried lamina was impregnated with three different impregnating agents. First, the citric acid had a concentration of 20%. The second impregnating agent was a polystyrene solution mixed with the catalyst potassium peroxydisulfate with a ratio of 1:0.01 (*v*/*v*), followed by a 5% boric acid solution. Sample preparation and the concentrations were based on prior studies [24,26]. Higher levels of concentration or catalyst ratio risked excessive degradation of wood polymers or unstable polymerization. Impregnation was conducted in a vacuum cylinder for 30 min, followed by pressing for 30 min at a pressure of 6.6 bar. This was the optimal condition; longer/higher pressures caused delamination or adhesive squeeze-out. Furthermore, the impregnated lamina was wrapped in aluminum foil and dried in an oven at 60 °C for 3 days. Next, weight percent gain (WPG) was calculated to determine the amount of impregnating agent that penetrates and deposits into the lamina.

### 2.3. Manufacture of Laminated Board

The steps of laminated board manufacturing refer to Hartono et al. [24] as follows: Jabon lamina was arranged into three layers with a size of 30 cm × 15 cm × 1.5 cm for length, width, and thickness. In the laminated composites, the growth rings of the laminae were oriented in a parallel direction to improve mechanical properties and dimensional stability. The laminated board was glued using isocyanate adhesive and hardener (85:15 *w*/*w*%), with an adhesive spread level of 280 g/m^2^ with double spread application. The ratio of 85:15 was selected according to the manufacturer’s recommendation for optimal curing [13]. The laminated board was then cold pressed using clamps on all sides for 24 h and conditioned for 10 days at room temperature before testing to achieve a state of equilibrium with the environment.

### 2.4. Testing and Evaluation of the Properties of the Laminated Board

All physical and mechanical properties tests were carried out with five replications for each treatment, while chemical analyses (FTIR, XRD, Py-GCMS) were performed in three to ensure reproducibility.

#### 2.4.1. Physical and Mechanical Testing

The properties of the laminated board, including physical and mechanical properties, were tested according to the standard of JAS 234:2003. The physical properties include density, moisture content, delamination, and weight percent gain (WPG). Mechanical properties were the modulus of elasticity (MOE), modulus of rupture (MOR), and shear strength (SS).

#### 2.4.2. Chemical Analysis

The chemical characterization of a bamboo laminated board was performed using Fourier Transform Infrared Spectroscopy (FTIR), Pyrolysis Gas Chromatography–Mass Spectrometry (Py-GCMS), and X-ray Diffraction (XRD). To detect the reaction between the impregnating agent and the Jabon wood, functional group analysis was conducted using FTIR (Perkin Elmer Spectrum Two) with an average of 16 scans at a resolution of 4 cm^−1^. Py-GCMS (Shimadzu GCMS-QP2020 NX, Shimadzu Corporation, Kyoto, Japan) was used to analyze changes in the chemical compounds in the laminated board, and XRD was used to observe variations in crystallinity.

### 2.5. Data Analysis

Analysis of variance (ANOVA) in Microsoft Excel 2010 was used to examine the data to assess the impact of the levels and changes in the variables. To find out whether groups had substantially different means at a 95% confidence level, mean comparisons were performed using Duncan’s test.

## 3. Results and Discussion

### 3.1. Physical Properties

**Figure 1:**
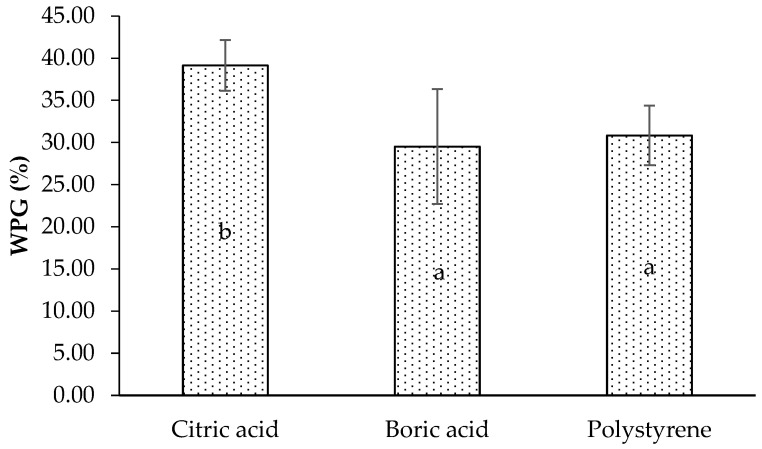
Weight percent gain of lamina with different impregnating agents. Letters of a and b indicate significant differences in statistical analysis.

The result showed that citric acid is the greatest penetrating agent for the wood, followed by polystyrene and boric acid (Figure 1). In this study, citric acid had the highest concentration among the three impregnating agents used. The high treatment concentration tends to cause a higher weight percent gain in the sample [27]. Rumbaremata et al. [28] reported that soaking Samama wood in citric acid solution with 5% and 10% resulted in weight gains of 3.46% and 14.36%, respectively. In addition, Basri et al. [29] also reported that treating laminated boards with citric acid concentrations of 20% and 40% resulted in weight gains of 25% and 28%, respectively. Similar results were obtained by Fidan and Adanur [20] using borax and boric acid; as the concentration increases, the amount of the substance that adheres to the wood increases. Higher solution concentration generates a steeper gradient between wood and solution, promoting stronger diffusion into cell walls [30].

The availability of chemical agents is greater at high concentrations, thus penetrating and filling the wood’s cell walls and voids. Differences in the concentration of impregnating agents in this study may influence weight percent gain (WPG), although these concentrations were optimized in prior research [24,26]. However, future studies should explore identical concentrations for more direct comparisons. Statistical analysis showed that impregnation treatment significantly affected WPG (*p* < 0.05). Based on Duncan’s multiple range test, the citric acid impregnating agent significantly differed from boric acid and polystyrene in the WPG of impregnated wood (shown by different letters a and b on the diagram). This confirms that differences in impregnating agent types influence the impregnated lamina’s WPG.

**Figure 2:**
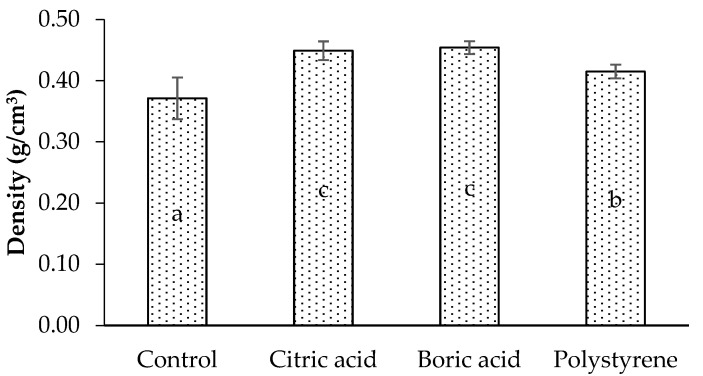
Density of laminated board with different impregnating agents. Letters of a, b, and c indicate significant differences in statistical analysis.

The density of the laminated board is shown in Figure 2. Compared to a control (untreated) board, a laminated board with impregnated lamina had a higher density. The control (untreated) laminated board density was 0.37 g/cm^3^, similar to the density of Jabon wood of 0.35 g/cm^3^ [3]. Using impregnated lamina in manufacturing, the Jabon laminated board had a higher density of 10–20% (0.41–0.45 g/cm^3^). This increase in the density of the laminated board is a result of the addition of weight by the impregnating agent remaining in each layer of wood. The density increases with weight, but the relative volume remains the same. The highest density of the laminated board is impregnated with citric acid and boric acid. Yusof et al. [31] treated bamboo with 5% boric acid by the immersion method for 24 h and showed an increase in density. According to Fidan and Adanur [20], because the boron compounds utilized in the impregnation process have salt characteristics, they increase the density. In addition, the amount of substance that adheres to the wood increases with the concentration of the boron compound. The analysis of variance showed that impregnation treatment significantly affected the density of the laminated board (*p* < 0.05). Duncan’s analysis shows that treated lamina with citric acid and boric acid produced laminated boards that significantly differed from control (untreated) (using untreated lamina) and those treated with polystyrene.

**Figure 3:**
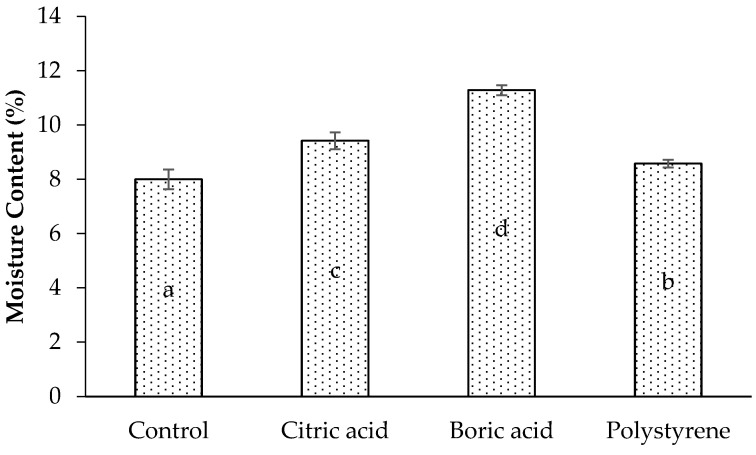
Moisture content of laminated board with different impregnating agents. Letters of a, b, c, and d indicate significant differences in statistical analysis.

A laminated board using impregnated lamina has higher moisture content (MC) than a control (untreated) board. A similar result was obtained by Nurhanifah et al. [32]. Despite being greater than the control (untreated), the MC of the treated lamina is still within the 15% maximum allowed by the JAS 234 standard range limit. The hygroscopic nature of impregnating agents probably caused this higher MC, so the treated laminated board absorbs more environmental moisture. The hygroscopicity of organic compounds such as citric acid was demonstrated by Han et al. [22], followed by a boric acid compound as a water-based preservative [31]. On the other hand, polystyrene is a hydrophobic material [22,23]. Hygroscopic material will absorb moisture from the environment more than hydrophobic material. This explains why treated polystyrene has a lower moisture content than other types (Figure 3). The ANOVA results on MC showed that impregnation of the lamina significantly affected the density of the laminated board produced. Furthermore, all the impregnating agents produced statistically different results.

Another substantial physical property is delamination properties, which are used to observe the quality of adhesion to water. This study showed no delamination (0%) in all laminated boards after immersion for 4 h in hot water. The result is better than the study of Hartono et al. [24] on a bamboo laminated board with a delamination percentage of 0 to 7.7. Based on ANOVA, the impregnation treatment in the lamina does not affect the laminated board. This means high-quality laminated boards are produced from Jabon wood glued with isocyanate adhesive using impregnated or untreated laminates. It is also necessary to take into account potential opposing or synergistic interactions with the isocyanate adhesive in addition to the effects of the impregnants individually. Boric and citric acids may inhibit the formation of urethane bonds by decreasing the availability of reactive hydroxyl groups, while polystyrene impregnation seems to improve adhesive wetting and stress transmission at the interface. Such interactions likely contributed to the observed differences in bonding strength and mechanical performance. FTIR analysis showed that the chemical interaction with the wood’s modified hydroxyl groups appears to have reduced the isocyanate (-NCO) functional groups, enhancing bond formation with the isocyanate adhesive. With a maximum value of 10%, this study’s delamination of laminated boards generally complies with the JAS 234 standard.

### 3.2. Mechanical Properties

**Figure 4:**
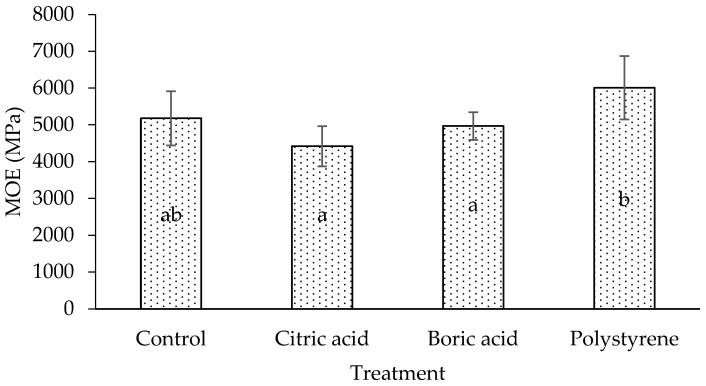
MOE of laminated board with different impregnating agents. Letters of a, b, and ab indicate significant differences in statistical analysis.

**Figure 5:**
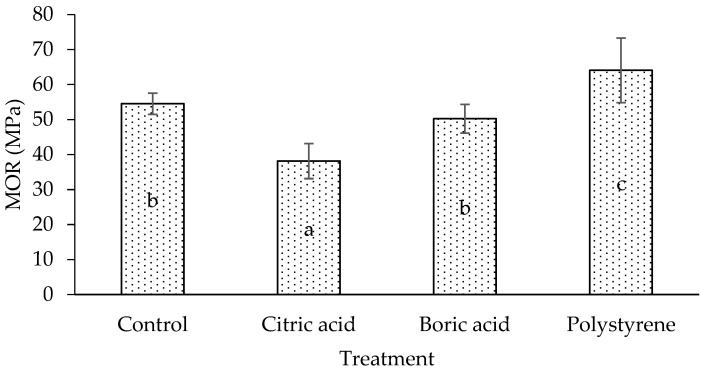
MOR of laminated board with different impregnating agents. Letters of a, b, and c indicate significant differences in statistical analysis.

The modulus of elasticity (MOE) and the modulus of rupture (MOR) of the laminated board are shown in Figure 4 and Figure 5. The results indicate that MOE and MOR follow similar trends, with citric acid, boric acid, and polystyrene showing the lowest to highest values, respectively. Compared to a control (untreated) board, treated citric acid and boric acid laminated boards have lower MOE and MOR. A similar result was obtained by a previous study [24,29]. Interestingly, although citric acid and boric acid increased the density (Figure 2) compared with the control, their mechanical properties (MOE, MOR, and shear strength) were lower. This apparent contradiction may be explained by the deposition of non-structural chemicals within the lumen and voids, which increases mass and thus density but does not reinforce the cell wall. Impregnation treatment in the lamina has a significant effect on the mechanical properties (MOE and MOR) of the laminated board (*p* < 0.05). Duncan’s multiple range test shows that the control (untreated) laminated board significantly differs from the treated laminated board, especially with polystyrene.

Due to their distinct chemical characteristics, polystyrene, citric acid, and boric acid affect wood’s mechanical properties differently. A study of citric acid pretreatment on sugarcane bagasse (SB) showed that when compared to raw SB, the acid pretreatment encouraged the fiber to peel and disintegrate. The partial removal of the lignin and hemicellulose fractions may have resulted in surface grooves in the pretreated material. The alterations brought about by the citric acid pretreatment of SB resulted in a partial rupture of the cell wall’s resistant structure [33]. This rupture of the cell wall leads to reduced strength and increased brittleness.

Meanwhile, a reduction in strength is caused by the boric acid impregnating agent, which contributes to its fixation interaction with wood and its chemical structure. Wood sugars undergo a hydrolytic depletion reaction with the impregnation materials that contain acidic chromium, leading to interaction with the cell wall components. This can somewhat weaken the wood because it interferes with hydrogen bonding [20]. Citric acid and boric acid likely caused partial hydrolysis of hemicellulose and amorphous carbohydrates, leading to localized weakening of the cell wall matrix. This effect reduces stress transfer efficiency and mechanical strength but does not represent a complete structural breakdown of cellulose. In contrast, impregnation with polystyrene generally improves the mechanical properties of wood by filling lumens and being tightly embedded in the wood’s hierarchical structure. In situ polymerization of hydrophobic styrene monomer within the wood cell walls and lumens improved the dimensional stability and mechanical qualities [22]. Thus, while polystyrene improves mechanical performance, citric and boric acids may decline if not carefully controlled (untreated), leading to structural alterations and chemical degradation. Although polystyrene has enhanced mechanical qualities, its environmental impact and persistence are issues; hence, future research should look into bio-based alternatives.

**Figure 6:**
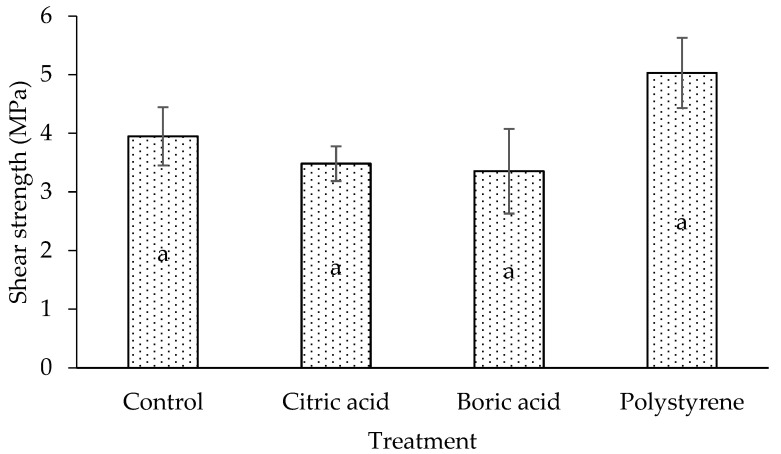
Shear strength of laminated board with different impregnating agents. The same letter (a) indicates no significant difference in statistical analysis.

The laminated board’s shear strength exhibits a pattern comparable to MOE and MOR (Figure 6). Although statistical analysis indicates that impregnation treatment does not affect shear strength, the laminated board impregnated with citric and boric acids has a lower shear strength than the control (untreated) laminated board. However, the shear strength of the resulting laminated board is increased when the lamina is impregnated with polystyrene. Polystyrene often increases shear strength by filling voids and reinforcing the cell structure, producing a composite-like effect that resists internal slippage. This mechanism is similar to that which was described in MOE and MOR. However, the hydrolysis of wood’s chemical components by citric and boric acids weakens the cell wall’s bonding, lowering the wood’s shear strength characteristics [20,22,33]. Overall, the increase in mechanical properties of the laminated board indicates that these impregnation treatments improve mechanical performance under load and stress distribution and change the plant’s chemical structure.

### 3.3. Chemical Characterization

**Figure 7:**
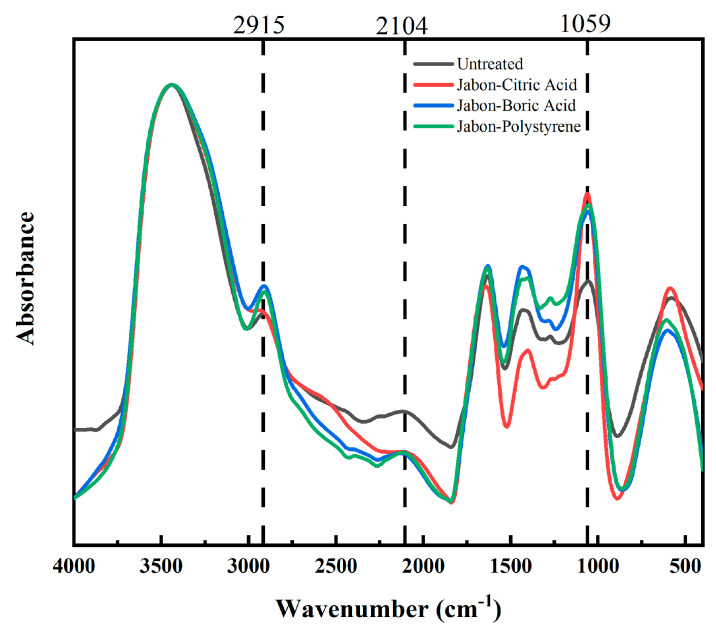
FTIR of laminated board with different impregnating agents.

The FTIR spectra revealed notable chemical changes in citric acid, boric acid, and polystyrene-impregnated Jabon laminated boards compared to untreated ones (Figure 7). In the first fingerprint region, there was an increase in the absorbance peak of 2915 cm^−1,^ which corresponds to C–H stretching vibrations in cellulose II, and can be found in the regions between 3000 and 2800 cm^−1^ [34], especially those impregnated with boric acid and polystyrene. It suggested a new aliphatic chain form or enhanced hydrocarbon polymer interaction with the lignocellulosic matrix in the impregnated laminated board.

The second region showed a significant decrease in the peak intensity at 2104 cm^−1^ in all impregnated laminated boards. According to Szanyi et al. [35], it is possible to attribute the 2104 cm^−1^ band to reactions involving NO^+^ that result in the formation of HCN. Various characteristics resulting from adsorbed N2O, isocyanate, and potentially organonitrile, as connected to the production of N2O, HNCO, and nitriles, are found in the broad band between 2200 and 2300 cm^−1^. The decrease in peak intensity at ~2104 cm^−1^ likely reflects the consumption of nitrogen-containing functional groups, including isocyanate (–NCO), nitrile (–C≡N), or isocyanurate species, through reactions with hydroxyl groups in the wood or side reactions with impregnation residues that reduce the number of reactive groups. This reduction should not be assigned exclusively to HCN, but more generally indicates chemical interactions that affect adhesive bonding performance.

In addition, all impregnated laminated boards showed an increase in the absorbance peak at 1059 cm^−1^, which was characterized by a carbohydrate region between 1200 and 890 cm^−1^ and linked to C–O stretching vibration in alcohol groups [36,37]. At the same time, Karacan and Soy [34] found broad C–O stretching at an absorbance peak of 1055 cm^−1^ of viscose rayon fibers. All the impregnated laminated boards showed an increase in the absorbance band, which was particularly noticeable in citric acid-impregnated laminated boards. According to [29,38], this is in line with esterification reactions that occur between hydroxyl groups and citric acid on wood polymers. A general increase in absorbance in some fingerprint regions in the impregnated samples confirms the success of the impregnation treatment.

**Figure 8:**
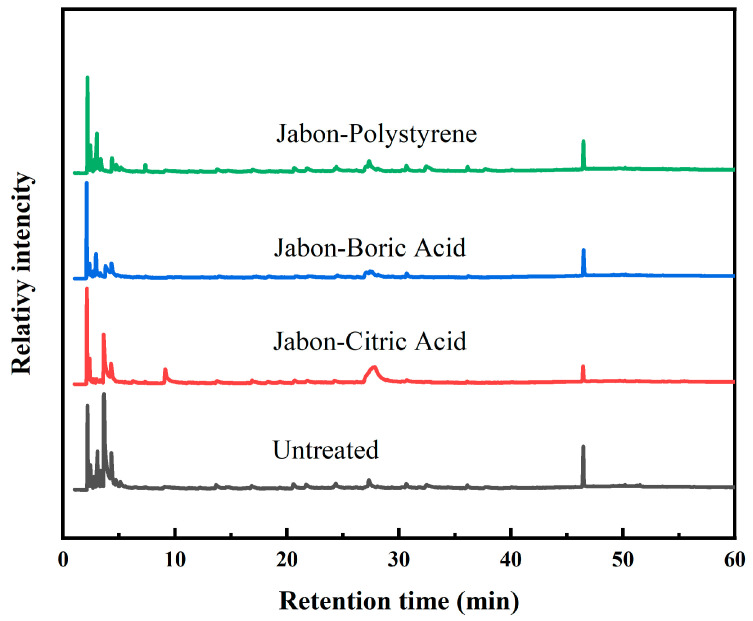
Py-GCMS of laminated board with different impregnating agents.

The Py-GCMS analysis demonstrates notable variations in peak intensity and distribution across treatments, indicating distinct pyrolysis degradation patterns (Figure 8). In the early retention time range (1–10 min), the untreated laminated board showed a high concentration of low-molecular-weight pyrolysis products, including acetic acid and furfural, typical degradation products of lignocellulosic material [39]. On the other hand, samples treated with citric acid showed reduced peak intensity in the early retention time, suggesting fewer hemicellulose-derived products due to ester bond formation. Furthermore, peaks around 10–25 min and near 46 min exhibited altered intensities, suggesting that hydroxyl groups in cellulose and hemicellulose have been partially esterified with citric acid. This outcome aligns with other studies showing that citric acid can function as a cross-linking agent, creating ester linkages that promote lowering hygroscopicity and increasing thermal stability [24,29].

Additionally, the boric acid-treated sample displays moderate suppression of peaks in the early retention time range, possibly due to borate complexation with polysaccharide chains, which restricts volatile release and heat degradation. This was confirmed in the Zhang et al. [40] study, which showed that boric acid affected the pyrolysis of lignocellulose by decreasing the volatile organic compound yield and composition. Subsequently, in the first 10 min retention time, the polystyrene-treated laminated board showed several peaks of low-molecular-weight pyrolysis products of lignocellulosic material. A styrene peak appears in the retention time of 7 min, but another study showed it at 13.45 min, with aromatic hydrocarbons being the only compounds found after polystyrene pyrolysis [41]. These findings show that by adding synthetic aromatic structures, polystyrene produces wood-rich aromatic hydrocarbons that contribute to hydrophobicity and modify its thermal degradation profile.

**Figure 9:**
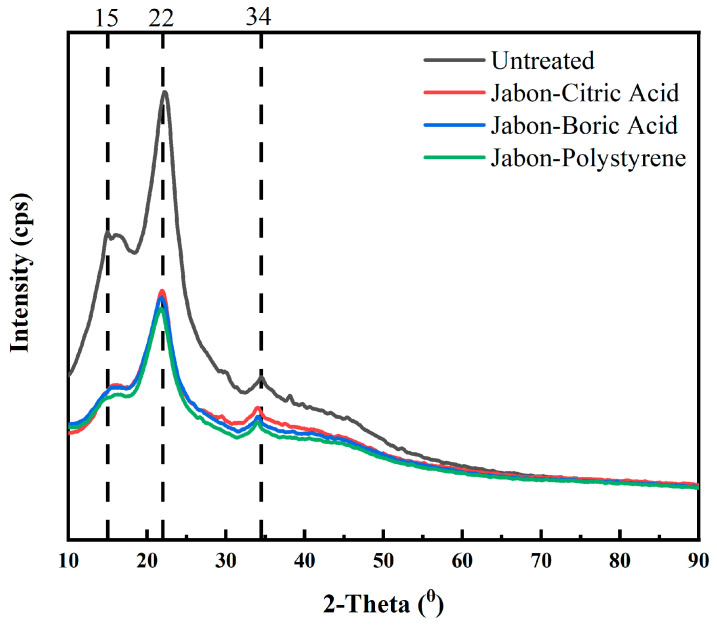
XRD of laminated board with different impregnating agents.

The X-ray diffraction (XRD) analysis showed the crystalline structure changes in the Jabon laminated board subjected to different chemical impregnation treatments (Figure 9). All samples exhibited a typical cellulose I diffraction pattern with dominant peaks observed near 2θ = 15°, 22°, and 34° [42]. With the highest intensity at 22°, the untreated laminated board showed a comparatively high degree of crystallinity (26.05%) and a native cellulose structure that was well-preserved. Conversely, there was a noticeable decrease in this peak intensity in the impregnated samples, suggesting that chemical modification influenced the cellulose microstructure. The crystallinity index of laminated boards impregnated with citric acid, boric acid, and polystyrene was 24.33%, 24.22%, and 24.64%, respectively. The observed decrease in the degree of crystallinity due to chemical impregnation treatments is closely associated with a reduction in mechanical properties. The citric acid- and boric acid-impregnated Jabon laminated board has lower mechanical properties. On the other hand, although the polystyrene-impregnated board experiences a decrease in the degree of crystallinity, its mechanical properties improve. This could be because the hydrophobic polymer of polystyrene physically fills the lumen spaces and cell wall cavities. Different results were shown by Hartono et al. [24], where the 22° peak intensity slightly increased after the bamboo laminated board was impregnated.

## 4. Conclusions

Impregnation treatment in Jabon lamina as a raw material for laminated board manufacturing significantly affects the laminated board’s physical and mechanical properties. Citric and boric acids increase density but reduce strength due to the partial hydrolysis of cell wall components, while polystyrene improves both strength and dimensional stability by reinforcing the wood structure. Practically, polystyrene was the most effective agent for enhancing strength and stability. However, its synthetic and non-biodegradable nature raises environmental concerns; furthermore, bio-based polymers could be considered alternatives in future research. FTIR, Py-GCMS, and XRD confirmed chemical modifications, including esterification (citric acid), while boric acid and polystyrene increased hydrocarbon polymer, with all treatments lowering crystallinity. This emphasizes that mechanical performance is not determined by crystallinity alone but also by polymer reinforcement effects.

## Data Availability

The data presented in this study are available on request from the corresponding author.

## References

[1] Ketut I., Pandit N., Nandika D., Darmawan W. (2011). Analisis Sifat Dasar Kayu Hasil Hutan Tanaman Rakyat. J. Ilmu Pertan. Indones..

[2] Rachmi Trisatya D., Maria Sulastiningsih I. (2019). Sifat papan partikel dari campuran kayu jabon dan bambu andong. J. Penelit. Has. Hutan.

[3] Anna N., Siregar I.Z., Supriyanto S., Sudrajat D.J., Karlinasari L. (2023). Physical, Mechanical, and Anatomical Properties of 12 Jabon (Neolamarckia Cadamba) Provenances Wood in Indonesia. Biodiversitas J. Biol. Divers..

[4] Yunianti A.D., Taskirawati I., Muin M., Sanusi D., Suhasman, Agussalim A. (2017). Teknologi Tepat Guna Peningkatan Ketahanan Kayu Terhadap Organisme Perusak Kayu Untuk Bahan Baku Kerajinan Berkualitas. J. Din. Pengabdi..

[5] Hadjib N., Abdurachman, Basri E. (2015). Karakteristik fisis dan mekanis glulam jati, mangium dan trembesi. J. Penelit. Has. Hutan.

[6] Wulandari F.T., Lestari D., Amin R. (2023). Analisis sifat fisika mekanika papan laminasi laminasi kayu kemiri (Analysis of the Physical and Mechanical Properties of Laminated Board Combination of Petung Bamboo Rajumas Wood and Candlenut Wood Laminated Board. J. Kehutan. Indones. Celeb..

[7] Purwanto D., Riset B., Standardisasi D., Banjarbaru I. (2011). Pembuatan Balok Dan Papan Dari Limbah Industri Kayu Board and Wood Block Making From Waste of Wood Industries. J. Ris. Ind..

[8] Wulandari F.T., Amin R., Raehanayati R. (2022). Karateristik Sifat Fisika Dan Mekanika Papan Laminasi Kayu Sengon Dan Kayu Bayur. Euler J. Ilm. Mat. Sains Teknol..

[9] Widiati K.Y. (2018). Karakteristik sifat fisika dan mekanika kayu lamina kombinasi jenis kayu sengon (*Paraserianthes falcataria* (L.) Nilsen) dan jenis kayu merbau (*Intsia* Spp.). ULIN J. Hutan Trop..

[10] Wijaya E., Manik P., Jokosisworo S. (2017). Analisa Kekuatan Tarik Dan Kekuatan Lentur Balok Laminasi Bambu Petung Dan Kayu Sengon Untuk Komponen Kapal Kayu. Tek. Perkapalan.

[11] Handayani S. (2016). Analisis pengujian struktur balok laminasi kayu sengon dan kayu kelapa. J. Tek. Sipil Perenc..

[12] Okuda S., Corpataux L., Muthukrishnan S., Wei K.H. Cross-Laminated Timber with Renewable and Fast Growing Tropical Species in South East Asia. Proceedings of the WCTE 2018: 2018 World Conference on Timber Engineering.

[13] Hendrik J., Hadi Y.S., Massijaya M.Y., Santoso A. (2016). Properties of Laminated Composite Panels Made from Fast-Growing Species Glued with Mangium Tannin Adhesive. BioResources.

[14] Sandberg D., Kutnar A., Mantanis G. (2017). Wood Modification Technologies—A Review. IForest.

[15] Schorr D., Boivin G., Stirling R. (2024). Treatments to Improve the Dimensional Stability of White Spruce Cladding. Wood Fiber Sci..

[16] Seydibeyoğlu M.Ö., Dogru A., Wang J., Rencheck M., Han Y., Wang L., Seydibeyoğlu E.A., Zhao X., Ong K., Shatkin J.A. (2023). Review on Hybrid Reinforced Polymer Matrix Composites with Nanocellulose, Nanomaterials, and Other Fibers. Polymers.

[17] Yu T., Soomro S.A., Huang F., Wei W., Wang B., Zhou Z., Hui D. (2020). Naturally or Artificially Constructed Nanocellulose Architectures for Epoxy Composites: A Review. Nanotechnol. Rev..

[18] Gomes M.G., Gurgel L.V.A., Baffi M.A., Pasquini D. (2020). Pretreatment of Sugarcane Bagasse Using Citric Acid and Its Use in Enzymatic Hydrolysis. Renew. Energy.

[19] Lee S.H., Tahir P.M., Lum W.C., Tan L.P., Bawon P., Park B.D., Al Edrus S.S.A.O., Abdullah U.H. (2020). A Review on Citric Acid as Green Modifying Agent and Binder for Wood. Polymers.

[20] Fidan M.S., Adanur H. (2019). Physical and Mechanical Properties of Wood Impregnated with Quebracho and Boron Compounds. Forestist.

[21] Marney D.C.O., Russell L.J. (2008). Combined Fire Retardant and Wood Preservative Treatments for Outdoor Wood Applications—A Review of the Literature. Fire Technol..

[22] Han X., Wang Z., Zhang Q., Pu J. (2020). An Effective Technique for Constructing Wood Composite with Superior Dimensional Stability. Holzforschung.

[23] Huan S., Liu G., Han G., Cheng W., Fu Z., Wu Q., Wang Q. (2015). Effect of Experimental Parameters on Morphological, Mechanical and Hydrophobic Properties of Electrospun Polystyrene Fibers. Materials.

[24] Hartono R., Muliani P.F., Sutiawan J., Amanda P., Sumardi I., Rofii M.N. (2025). Properties of Laminated Board from Belangke Bamboo (Gigantochloa Pruriens) Modified with Citric Acid, Boric Acid, and Polystyrene. Wood Mater. Sci. Eng..

[25] Rowell R., Pettersen R., Tshabalala M. (2012). Handbook of Wood Chemistry and Wood Composites: Cell Wall Chemistry.

[26] Hartono R., Sutiawan J., Kusumah S.S., Tarmadi D., Wikantyoso B., Himmi S.K., Yusuf S., Zulfiana D., Roseley A.S.M., Abu F. (2025). Belanke Bamboo (Gigantochloa Pruriens) Laminated Board Modified with Polystyrene, Citric, and Boric Acid: Resistance from Terminte and Decay Attacks. BioResources.

[27] Basri E., Balfas J. (2014). Impregnasi ekstrak jati dan resin pada kayu jati cepat tumbuh dan karet. J. Penelit. Has. Hutan.

[28] Rumbaremata A., Cahyono T.D., Darmawan T., Kusumah S.S., Akbar F., Dwianto W. (2021). Peningkatan Kerapatan Kayu Samama Melalui Pre-Kompresi Asam Sitrat (Density Improvement of Samama Wood by Pre-Compression of Citric Acid). J. Ilmu Teknol. Kayu Trop..

[29] Basri E., Hanifah N., Martha R., Rahayu I.S., Mubarok M., Darmawan W., Gérardin P. (2022). Effect of Citric Acid and Benzophenone Tetracarboxyclic Acid Treatments on Stability, Durability, and Surface Characteristic of Short Rotation Teak. Forests.

[30] Tanaka S., Seki M., Miki T., Shigematsu I., Kanayama K. (2016). Solute Diffusion into Cell Walls in Solution-Impregnated Wood under Conditioning Process II: Effect of Solution Concentration on Solute Diffusion. J. Wood Sci..

[31] Yusof N.M., Hua L.S., Tahir P.M., James R.M.S., Al-Edrus S.S.O., Dahali R., Roseley A.S.M., Fatriasari W., Kristak L., Lubis M.A.R. (2023). Effects of Boric Acid Pretreatment on the Properties of Four Selected Malaysian Bamboo Strips. Forests.

[32] Nurhanifah, Hermawan D., Hadi Y.S., Arsyad W.O.M., Abdillah I.B. (2020). Shear Strength and Subterranean Termite Resistance of Polystyrene Impregnated Sengon (Falcataria Moluccana) Glulam. IOP Conf. Ser. Mater. Sci. Eng..

[33] Gonçalves F.G., Paes J.B., Lopez Y.M., de Alcântara Segundinho P.G., de Oliveira R.G.E., Fassarella M.V., Brito A.S., Chaves I.L.S., Martins R.S.F. (2021). Resistance of Particleboards Produced with Ligno-Cellulosic Agro-Industrial Wastes to Fungi and Termites. Int. Biodeterior. Biodegrad..

[34] Karacan I., Soy T. (2013). Structure and Properties of Oxidatively Stabilized Viscose Rayon Fibers Impregnated with Boric Acid and Phosphoric Acid Prior to Carbonization and Activation Steps. J. Mater. Sci..

[35] Szanyi J., Kwak J.H., Moline R.A., Peden C.H.F. (2004). Adsorption, Coadsorption, and Reaction of Acetaldehyde and NO_2_ on Na-Y,FAU: An in Situ FTIR Investigation. J. Phys. Chem. B.

[36] Kostryukov S.G., Matyakubov H.B., Masterova Y.Y., Kozlov A.S., Pryanichnikova M.K., Pynenkov A.A., Khluchina N.A. (2023). Determination of Lignin, Cellulose, and Hemicellulose in Plant Materials by FTIR Spectroscopy. J. Anal. Chem..

[37] Javier-Astete R., Jimenez-Davalos J., Zolla G. (2021). Determination of Hemicellulose, Cellulose, Holocellulose and Lignin Content Using FTIR in *Calycophyllum spruceanum* (Benth.) K. Schum. and *Guazuma crinita* Lam. PLoS ONE.

[38] Lee S.H., Ashaari Z., Ang A.F., Abdul Halip J., Lum W.C., Dahali R., Halis R. (2018). Effects of Two-Step Post Heat-Treatment in Palm Oil on the Properties of Oil Palm Trunk Particleboard. Ind. Crops Prod..

[39] Chen W.H., Wang C.W., Ong H.C., Show P.L., Hsieh T.H. (2019). Torrefaction, Pyrolysis and Two-Stage Thermodegradation of Hemicellulose, Cellulose and Lignin. Fuel.

[40] Zhang J., Koubaa A., Xing D., Wang H., Wang Y., Liu W., Zhang Z., Wang X., Wang Q. (2020). Conversion of Lignocellulose into Biochar and Furfural through Boron Complexation and Esterification Reactions. Bioresour. Technol..

[41] Hassan E.B., Elsayed I., Eseyin A. (2016). Production High Yields of Aromatic Hydrocarbons through Catalytic Fast Pyrolysis of Torrefied Wood and Polystyrene. Fuel.

[42] Montoya-Escobar N., Ospina-Acero D., Velásquez-Cock J.A., Gómez-Hoyos C., Serpa Guerra A., Gañan Rojo P.F., Vélez Acosta L.M., Escobar J.P., Correa-Hincapié N., Triana-Chávez O. (2022). Use of Fourier Series in X-Ray Diffraction (XRD) Analysis and Fourier-Transform Infrared Spectroscopy (FTIR) for Estimation of Crystallinity in Cellulose from Different Sources. Polymers.

